# The Impact of Moderate to High Intensity Physical Activity on Sleep Health in Cancer Survivors

**DOI:** 10.1002/cam4.71546

**Published:** 2026-02-01

**Authors:** Grace E. Markey, Julie J. Ruterbusch, Tara E. Baird, Jennifer L. Martin, Ann G. Schwartz, David G. Finlay, Trey Timban, Matthew R. Trendowski, M. Safwan Badr, Kerri Winters‐Stone, Jennifer L. Beebe‐Dimmer

**Affiliations:** ^1^ Department of Oncology Wayne State University School of Medicine Detroit Michigan USA; ^2^ Barbara Ann Karmanos Cancer Institute Detroit Michigan USA; ^3^ Department of Medicine David Geffen School of Medicine at the University of California, Los Angeles Los Angeles California USA; ^4^ Geriatric Research, Education and Clinical Center VA Greater Los Angeles Healthcare System Los Angeles California USA; ^5^ Division of Pulmonary, Allergy, Critical Care and Sleep Medicine Wayne State University School of Medicine Detroit Michigan USA; ^6^ Division of Oncologic Sciences Oregon Health & Science University School of Medicine Portland Oregon USA; ^7^ Oregon Health & Science University, Knight Cancer Center Portland Oregon USA

**Keywords:** exercise, insomnia, quality of life, sleep health, sleep score

## Abstract

**Background:**

Sleep disturbances are common among cancer survivors and negatively impact quality of life. Regular moderate‐ to high‐intensity physical activity may provide a cost‐effective, low‐risk alternative strategy to improve sleep.

**Methods:**

Data collected as part of two distinct studies, the Detroit Research On Cancer Survivors (ROCS) cohort and the CrossFit And Physical Activity: A Better Life Experience (CAPABLE) High‐Intensity Interval Training (HIIT) trial, were analyzed to evaluate the association between participation in moderate‐ to high‐intensity physical activity and sleep health. Sleep health was assessed using the Insomnia Severity Index (ISI), Pittsburgh Sleep Quality Index (PSQI), and Epworth Sleepiness Scale (ESS).

**Results:**

Among Detroit ROCS cohort members who completed the supplemental sleep survey at baseline and/or follow‐up (*n* = 3022), those meeting 2012 American Cancer Society (ACS) physical activity guidelines reported sleep outcomes compared with inactive participants, including lower ISI scores (4.5 vs. 5.9, *p* < 0.001), lower ESS scores (5.6 vs. 6.6, *p* < 0.001), and lower PSQI (6.3 vs. 7.9, *p* < 0.001). In the CAPABLE trial (*n* = 73), ISI scores improved from 4.5 at baseline to 3.4 at exit (*p* < 0.001), while PSQI scores showed more modest improvement (6.1 to 5.4, *p* = 0.063). ESS scores remained unchanged (5.4 to 5.2, *p* = 0.708).

**Conclusions:**

These findings support the role of moderate‐ to high‐intensity physical activity in improving sleep health in a diverse cancer survivor population. Future research should further refine current methodologies to maximize benefit to survivors and implementation science to increase uptake and promote adherence to evidence‐based guidelines.

## 1 Introduction

As cancer survival rates improve, attention has increasingly turned to the quality of survivorship. There are currently over 18 million cancer survivors in the United States—a number expected to exceed 22 million by 2030 due to advances in detection, treatment, and aging demographics [1, 2]. Despite these gains, racial disparities in outcomes remain prominent. Black cancer survivors continue to experience disproportionate challenges, including lower survival rates and greater post‐treatment symptom burden [3]. These disparities stem from systemic and individual factors affecting screening, treatment access, clinical trial participation, and post‐treatment care [3]. With increasing survivorship, efforts must focus on mitigating the physical and psychological effects of both the disease and its treatment.

One persistent and often under addressed challenge is sleep disruption. Sleep plays a vital role in physiological restoration—supporting cognitive function, emotional regulation, cardiovascular and metabolic health, and cellular repair [4]. Unfortunately, poor sleep is common and linked to adverse health consequences [5]. Cancer survivors face persistent challenges, with fatigue, insomnia, neuropathy, and pain significantly affecting their quality of life [6]. Nearly 80% of patients report sleep difficulties during treatment, and roughly 20% continue to experience poor sleep quality up to a decade post‐diagnosis [7, 8, 9]. These outcomes underscore the need to identify modifiable predictors of sleep health in this population—particularly among Black survivors, who are underrepresented in both sleep and survivorship research.

Evidence is mounting to suggest that physical activity may improve sleep quality in the general population and among cancer survivors. Regular exercise independently enhances quality of life and reduces post‐treatment fatigue, anxiety, and depression in cancer patients [10, 11, 12, 13, 14, 15, 16, 17, 18, 19]. However, the relationship between physical activity and sleep quality among Black cancer survivors remains underexplored. Our first aim is to examine whether regular physical activity is associated with better sleep quality in Black cancer survivors, using data from the Detroit ROCS cohort.

Our second aim is to evaluate whether a structured HIIT exercise intervention improves sleep quality in a diverse group of cancer survivors. Within this aim, we examine changes in validated sleep metrics (PSQI, ISI) and HRQOL following the intervention. In 2012, the American College of Sports Medicine (ACSM) and American Cancer Society (ACS) convened experts to establish best‐practice exercise guidelines for cancer survivors. Both organizations recommended avoiding inactivity and returning to daily activities as soon as possible, aiming for at least 150 min of moderate‐intensity or 75 min of vigorous‐intensity exercise per week, plus muscle‐strengthening exercises twice weekly [20, 21]. HIIT is a method that alternates quick bursts of exercise performed at maximal effort with either rest or low‐intensity movement. It engages both anaerobic and aerobic energy systems, enhancing cardiovascular fitness and metabolic function. Research on HIIT in cancer survivors, though limited, suggests it is well‐tolerated and improves quality of life [22, 23, 24, 25, 26, 27, 28, 29, 30, 31, 32]. CrossFit, a high‐intensity functional training modality based on HIIT principles, may confer additional benefits through its emphasis on community, mental resilience, and whole‐body strength. Thus, the novelty of this investigation lies both in its exploration of the HIIT method on sleep outcomes and its focus on a high‐risk and medically underserved population.

## 2 Methods and Materials

### 2.1 Study Population

#### 2.1.1 The Detroit Research on Cancer Survivors (ROCS) Cohort

Detroit ROCS is one of the largest studies ever conducted exclusively among Black cancer survivors to understand the multiplex causes for the poorer outcomes observed in this population after a diagnosis. Initiated in 2017, Detroit ROCS has enrolled more than 5000 Black survivors diagnosed with female breast, prostate, colorectal, lung, endometrial cancer at any age, or any other cancer diagnosed before age 50 years (considered early‐onset). Full participation in Detroit ROCS involves completing a baseline questionnaire, donating a blood or saliva sample for genomic evaluation, consent to tumor specimen and access to medical records as well as contact for annual follow‐up and future studies. The baseline and follow‐up surveys gather information on sociodemographic characteristics, health behaviors including physical activity, medical history including cancer treatment(s), treatment‐related toxicities, cancer progression and recurrence, family history of cancer, HRQOL, anxiety and depression [32]. In 2020, an administrative supplement to Detroit ROCS was awarded to evaluate sleep health in participants. The sleep supplement, added to both the baseline and annual follow‐up surveys, consisted of three validated sleep instruments, the Pittsburgh Sleep Quality Index (PSQI), [33] the Insomnia Severity Index (ISI) [34] and the Epworth Sleepiness Scale (ESS) [35].

#### 2.1.2 CrossFit and Physical Activity: A Better Life Experience (CAPABLE)

CAPABLE is a twelve‐week, single‐arm exercise intervention (Clinical Trials ID: NCT03750981) that introduces cancer survivors to CrossFit, a group training sport that mixes high‐intensity interval and strength building exercises. Study eligibility criteria include (1) age 18 and older at the time of program recruitment; (2) diagnosed with any invasive cancer; (3) cleared by a medical professional to participate in the program; (4) not currently participating in a regular fitness program; (5) no wide‐spread metastatic disease to brain and/or bone; and (6) available transportation to performance sites. Written medical clearance by a treating physician (oncologist or primary cancer physician) was required prior to enrollment. Study informed consent and a liability waiver for the performance sites were also completed prior to the start of the intervention.

The intervention consisted of one week of demonstration of basic functional movements and exercises, determination of appropriate scaling options for each individual participant and baseline testing. One unique characteristic of this intervention is that the exercises, including the baseline performance tests, are infinitely and individually scalable. This allows participants to achieve a similar physical stimulus regardless of their fitness level. For the remaining 11 weeks, all patients participated in three 60‐min sessions led by a certified CrossFit coach. Intervention adherence was defined as having attended at least 75% of all sessions with end of study testing during the last week of the intervention. The baseline and end of study testing for participants relied on pre‐determined scaled exercises, performed under the supervision of the coach and one additional evaluator, using a variety of standard tests commonly used in CrossFit programming. The performance tests were focused on evaluating a participant's (1) functional movement, (2) flexibility, (3) strength, and (4) aerobic capacity. All participants completed a survey at baseline, 6 weeks and at the end of the intervention to collect information including HRQOL measured by the FACT‐G and emotional response to exercise [36]. The addition of sleep questionnaire data to the intervention in 2020 coincided with the addition of the sleep supplement to Detroit ROCS, thus the sleep study in CAPABLE is considered pilot in nature. All participants in both the Detroit ROCS and CAPABLE studies provided informed consent, and the study protocols, surveys, and all documents were reviewed and approved by the Institutional Review Board at Wayne State University (Detroit, MI), IRB #080418MP2F and #050417M1F.

### 2.2 Sleep Scales

The Insomnia Severity Index (ISI) is a tool used to evaluate the nature, severity, and consequences of sleep disturbances [34]. It consists of 7 items with scores ranging from 0 to 28, where higher scores signify greater levels of insomnia. Scores were calculated for all participants who responded to at least three questions. The ISI is commonly used and considered a reliable instrument of insomnia with Cronbach alpha values between 0.74 and 0.91 [34, 37]. A score of 0–7 is indicative of no insomnia, (8–14) subthreshold insomnia, with moderate or severe (or clinical) insomnia with scores above 14. The ESS questionnaire measures general daytime sleepiness [38]. Participants rate the likelihood of dozing off or falling asleep in eight different scenarios in the past week using a Likert format. Scores range from 0 to 24, with higher scores generally reflecting greater daytime sleepiness. Like the ISI, the ESS is known for its reliability [35, 39]. However, unlike the ISI, the ESS score requires a response to all 8 items. Scores are then dichotomized to low (considered a “normal” level of sleepiness) or high (indicating “excessive sleepiness”). Participant responses to a modified version of the Pittsburgh Sleep Quality Index (PSQI) create a composite of overall sleep quality over the past week [33, 40]. The PSQI collects self‐assessed sleep quality, sleep latency, sleep duration, habitual sleep efficiency, sleep disturbances, use of sleeping medication, and daytime dysfunction. The component scores are combined to produce a total score, with higher scores indicating poorer sleep quality. PSQI is widely used with demonstrated validity.

### 2.3 Statistical Analysis

All analyses were performed using SAS software (Cary, NC) version 9.4 and an alpha < 0.05 was considered statistically significant. For Detroit ROCS, select demographic (sex, age at diagnosis, age at survey, education, marital status, and income), clinical (cancer site, SEER summary stage, and comorbidity count), and quality of life (PROMIS Anxiety and Depression scores categorized as none/mild symptoms versus moderate to severe symptoms) variables for survivors who completed the sleep supplement questionnaire were summarized using counts and percentages. Self‐reported physical activity was collected using a modified version of the short form of the International Physical Activity Questionnaire (IPAQ‐SF) [41]. The IPAQ‐SF collects information on both the intensity and duration of physical activity, and we used this information to create 3 categorical variables: meeting 202 ACS recommendations for physical activity (≥ 150 min per week of moderate to vigorous activity), some exercise but not meeting ACS recommendations (> 0 min and < 150 min per week of moderate to vigorous activity), and no reported physical activity (0 min of moderate to vigorous physical activity). Total number of exercise minutes per week was calculated by using the midpoint within each of the categorical responses for duration and frequency of both moderate and vigorous activity, and then summed to determine total. We elected to use the 2012 recommendations despite having more recent guidance given the timing of the cancer diagnoses of participants, as most were diagnosed before the 2019/2022 revisions. The mean, standard deviation, median, and range were calculated for each sleep measure by physical activity group. Differences in group means were evaluated using analysis of variance. In addition, the distribution of sleep scores categorized into clinically meaningful variables were compared by physical activity group using either the Mantel–Haenszel chi‐square test or the Cochran–Armitage test for trend, as applicable. Logistic regression was used to estimate the odds and 95% confidence intervals for reporting clinical insomnia (based on ISI score), excessive daytime sleepiness (based on ESS score), and poor quality (based on PSQI score) with meeting ACS physical activity recommendations. Model 1 was adjusted for variables chosen a priori based on previous publications that showed a significant association with both sleep, physical activity, and quality of life: education, income, and comorbidity count [32, 42]. Model 2 was adjusted for variables significantly associated with the 3 sleep measures in univariate analyses: cancer site/sex, age at diagnosis (10‐year age groups), marital status, income, comorbidity count, moderate to severe anxiety symptoms, and moderate to severe depression symptoms.

For CAPABLE, the primary outcome for this study was change over time with the introduction of the intervention on measures of sleep health (ISI, PSQI, and ESS). The distribution of age at enrollment, gender, self‐reported race, and cancer site was summarized using counts and percentages. Summary statistics (mean, standard deviation, median, and range) were calculated for each continuous sleep measure at baseline and exit and compared using paired *t*‐tests. In addition, the categorical variables for each sleep measure were compared between baseline and study exit using McNemar's test.

## 3 Results

### 3.1 Detroit ROCS Cohort

A total of 3022 Detroit ROCS participants both completed the sleep supplement and reported physical activity levels at either study enrollment or during follow‐up. Of these, 44.2% identified as male and 55.8% as female, with a majority aged 60 years or older at the time of survey (68%). The most common cancer diagnoses were breast (38.0%) and prostate (35.9%) (Table 1). Approximately 37% of participants reported annual household incomes below $20,000, and 64.6% had local stage cancer at diagnosis, while 27.6% had regional and 7.1% had distant stage disease.

**TABLE 1:** Select demographic and clinical variables for cancer survivors enrolled in the Detroit ROCS Cohort that completed the sleep supplement questionnaire.

	*N*	%
Total	3022	
Sex		
Male	1337	44.2%
Female	1685	55.8%
Age at cancer diagnosis (years)		
< 50	550	18.2%
50–59	828	27.4%
60–69	1111	36.8%
70+	533	17.6%
Marital status		
Married or equivalent	1165	39.0%
Widowed	310	10.4%
Divorced or separated	755	25.3%
Never married	759	25.4%
Income (household)		
< $20,000	1018	36.9%
$20,000–39,999	617	22.3%
$40,000–59,999	417	15.1%
$60,000–79,999	282	10.2%
≥ $80,000	427	15.5%
Cancer site		
Breast	1148	38.0%
Colorectal	202	6.7%
Endometrial	134	4.4%
Lung	296	9.8%
Prostate	1084	35.9%
Other	158	5.2%
SEER summary stage		
Local	1951	64.6%
Regional	835	27.6%
Distant	216	7.1%
Unknown	20	0.7%
Comorbidity count^a^		
None	239	8.0%
1	437	14.7%
2	527	17.7%
3	580	19.5%
4 or more	1188	40.0%
PROMIS anxiety symptoms		
None to mild	2645	92.3%
Moderate to severe	221	7.7%
PROMIS depression symptoms		
None to mild	2759	95.1%
Moderate to severe	141	4.9%

^a^ Self‐reported comorbidities include heart problems, COPD or emphysema, stroke, high blood pressure, high cholesterol, arthritis, hepatitis, diabetes, fracture (over the age of 50), thyroid problems, depression, and obesity.

Physical activity was associated with significantly better sleep outcomes (all *p* < 0.001; Table 2). Participants meeting ACS physical activity recommendations had: lower mean ISI scores (4.5 ± 5.0) compared to those not meeting recommendations (5.2 ± 5.4, *p* < 0.001) or reporting no physical activity (5.9 ± 6.0, *p* < 0.001). A higher proportion of participants without clinically significant insomnia (77.0%) compared to those not meeting recommendations (73.0%, *p* < 0.001) or inactive (68.0%, *p* < 0.001). Lower prevalence of clinical insomnia (5.0%) compared to those not meeting recommendations (7.5%, *p* < 0.001) or inactive (9.7%, *p* < 0.001). Lower mean ESS scores (5.6 ± 4.1) compared to those not meeting recommendations (6.0 ± 4.3, *p* < 0.001) or inactive (6.6 ± 4.7, *p* < 0.001). A higher proportion of participants with normal levels of daytime sleepiness (88.2%) compared to those not meeting recommendations (86.2%, *p* < 0.001) or inactive (81.7%, *p* < 0.001). Lower mean PSQI scores (6.3 ± 4.1) compared to those not meeting recommendations (7.0 ± 4.4, *p* < 0.001) or inactive (7.9 ± 4.5, *p* < 0.001).

**TABLE 2:** Sleep health measures by self‐reported moderate and vigorous physical activity minutes categorized based on ACS recommendations in Detroit ROCS cohort.

	Meets ACS recommendation		Less than ACS recommendation		No physical activity		*p*
Insomnia Severity Index (ISI)							
Mean (SD)	4.5 (5.0)		5.2 (5.4)		5.9 (6.0)		< 0.001
Median (range)	3 (0–28)		3 (0–28)		4 (0–28)		
No clinically significant insomnia (≤ 7)	741	77.0%	769	73.0%	666	68.0%	< 0.001
Subthreshold insomnia (8–14)	173	18.0%	206	19.5%	219	22.3%	
Clinical insomnia (15+)	48	5.0%	79	7.5%	95	9.7%	
Epworth Sleepiness Scale (ESS)							
Mean (SD)	5.6 (4.1)		6.0 (4.3)		6.6 (4.7)		< 0.001
Median (range)	5 (0–24)		5 (0–24)		6 (0–24)		
“Normal” daytime sleepiness (0–10)	816	88.2%	836	86.2%	742	81.7%	< 0.001
“Excessive Daytime Sleepiness” (11–24)	109	11.8%	134	13.8%	166	18.3%	
Pittsburgh Sleep Quality Index (PSQI)							
Total score							
Mean (SD)	6.3 (4.1)		7.0 (4.4)		7.9 (4.5)		< 0.001
Median (range)	5 (0–21)		6 (0–20)		7 (0–19)		
Good sleep quality (0–5)	365	50.9%	348	43.7%	243	34.8%	< 0.001
Poor sleep quality (6–21)	352	49.1%	449	56.3%	456	65.2%	
Sleep efficiency							
Mean (SD)	2.1 (1.9)		2.3 (2.0)		2.6 (2.0)		< 0.001
Median (range)	2 (0–6)		2 (0–6)		2 (0–6)		
Perceived sleep quality							
Mean (SD)	2.5 (2.1)		2.8 (2.2)		3.2 (2.4)		< 0.001
Median (range)	2 (0–9)		2 (0–9)		3 (0–9)		
Daily disturbances							
Mean (SD)	1.7 (1.1)		2.0 (1.2)		2.2 (1.3)		< 0.001
Median (range)	2 (0–6)		2 (0–6)		2 (0–6)		

In multivariable models adjusting for cancer site and sex, age at diagnosis, marital status, income, comorbidity count, moderate to severe anxiety symptoms, and moderate to severe depression symptoms, meeting ACS physical activity recommendations was associated with significant reductions in the odds of clinical insomnia (adjusted OR (aOR) = 0.66, 95% CI 0.43–0.99), excessive daytime sleepiness (aOR = 0.65, 95% CI 0.52–0.92), and poor sleep quality (aOR = 0.61, 95% CI 0.48–0.78) (Table 3; Model 2).

**TABLE 3:** Associations between meeting ACS physical activity recommendations and clinically meaningful sleep categories.

	Unadjusted		Model 1		Model 2	
	OR	95% CI	OR	95% CI	OR	95% CI
Outcome = Clinical insomnia						
No physical activity	1.00		1.00		1.00	
Less than ACS recommendation	0.75	0.55–1.03	0.81	0.58–1.14	0.87	0.60–1.24
Meets ACS recommendation	0.50	0.35–0.72	0.65	0.44–0.96	0.66	0.43–0.99
Outcome = Excessive daytime sleepiness						
No physical activity	1.00		1.00		1.00	
Less than ACS recommendation	0.70	0.54–0.90	0.72	0.55–0.94	0.69	0.48–0.88
Meets ACS recommendation	0.59	0.45–0.76	0.71	0.53–0.94	0.65	0.52–0.92
Outcome = Poor sleep quality						
No physical activity	1.00		1.00		1.00	
Less than ACS recommendation	0.69	0.56–0.86	0.74	0.59–0.93	0.74	0.58–0.94
Meets ACS recommendation	0.52	0.42–0.64	0.60	0.47–0.75	0.61	0.48–0.78

*Note:* Model 1 adjusted for education, income, and comorbidity count. Model 2 adjusted for cancer site and sex, age at diagnosis (10‐year age groups), marital status, income, comorbidity count, moderate to severe anxiety symptoms, and moderate to severe depression symptoms.

Abbreviations: CI, confidence interval; OR, odds ratio.

### 3.2 CAPABLE Trial

Among the CAPABLE trial participants with complete sleep data, the mean age at time of participation was 60.8 years (SD = 10.8) with an age range of 29–82 years. The majority were female (78%), with 60% identifying as Black/African American (Table 4). An analysis of pre‐ and post‐intervention sleep assessments demonstrated significant improvements in insomnia severity. ISI scores decreased from a mean of 4.5 (SD = 3.7) at baseline to 3.4 (SD = 3.1) at exit (*p* < 0.001), with the proportion of participants without clinically significant insomnia increasing from 72.6% to 87.7% (Table 5). ESS scores remained stable, with no significant change from baseline (5.4 ± 4.2) to exit (5.2 ± 4.2) (*p* = 0.708), with the proportion of participants with normal levels of daytime sleepiness slightly decreased from 91.4% to 87.1%. While overall PSQI scores suggested some improvement (6.1 ± 3.8 at baseline vs. 5.4 ± 3.5 at exit), this change did not reach statistical significance (*p* = 0.063). However, daily disturbances, a subcomponent of the PSQI, significantly decreased from a mean of 2.1 (SD = 1.2) to 1.8 (SD = 1.1) (*p* = 0.007), suggesting potential benefits in reducing day‐to‐day sleep disruptions.

**TABLE 4:** Characteristics of CAPABLE study participants in sleep study.

	*N*	%
Total	73	
Age at enrollment (years)		
Mean (std)	60.8 (10.8)	
Median (range)	29–82	
Sex		
Male	16	22%
Female	57	78%
Race		
White	28	38%
African American	44	60%
Other	1	1%
Cancer site		
Breast	40	55%
Prostate	8	11%
Endometrial	6	8%
Multiple myeloma	5	7%
All others	14	19%
Comorbidity count^a^		
None	6	8%
1	16	22%
2	21	29%
3	10	14%
4 or more	20	27%

^a^ Self‐reported comorbidities include heart problems, COPD or emphysema, stroke, high blood pressure, high cholesterol, arthritis, hepatitis, diabetes, fracture (over the age of 50), thyroid problems, depression, and obesity.

**TABLE 5:** Sleep health measures at baseline and exit surveys among CAPABLE sleep study participants.

	Baseline		Exit		*p*
Insomnia Severity Index (ISI)					
Mean (SD)	4.5 (3.7)		3.4 (3.1)		< 0.001
Median (range)	4 (0–13)		3 (0–12)		
No clinically significant insomnia (≤ 7)	53	72.6%	64	87.7%	0.005
Subthreshold insomnia (8–14)	20	27.4%	9	12.3%	
Clinical insomnia (moderate or severe) (15+)	0	—	0	—	
Epworth Sleepiness Scale (ESS)					
Mean (SD)	5.4 (4.2)		5.2 (4.2)		0.708
Median (range)	5 (0–19)		4 (0–20)		
“Normal” level of daytime sleepiness (0–10)	64	91.4%	61	87.1%	0.180
“Excessive Daytime Sleepiness” (11–24)	6	8.6%	9	12.9%	
Pittsburgh Sleep Quality Index (PSQI)					
Total score					
Mean (SD)	6.1 (3.8)		5.4 (3.5)		0.063
Median (range)	6 (0–15)		5 (0–14)		
Good sleep quality (0–5)	27	46.6%	34	58.6%	0.090
Poor sleep quality (6–21)	31	53.4%	24	41.4%	
Sleep efficiency					
Mean (SD)	1.8 (1.8)		1.6 (1.7)		0.515
Median (range)	1 (0–6)		1 (0–6)		
Perceived sleep quality					
Mean (SD)	2.5 (2.0)		2.2 (1.9)		0.191
Median (range)	2 (0–9)		2 (0–8)		
Daily disturbances					
Mean (SD)	2.1 (1.2)		1.8 (1.1)		0.007
Median (range)	2 (0–5)		2 (0–5)		

## 4 Discussion

The findings from this study provide important insights into the role of moderate‐ and high‐intensity physical activity in improving sleep health in a large and minority‐focused cancer survivor population, as demonstrated by the complementary results from the Detroit ROCS and CAPABLE studies. The Detroit ROCS cohort showed that meeting ACS physical activity guidelines was associated with significantly better sleep outcomes, including lower insomnia severity, reduced daytime sleepiness, and improved overall sleep quality. This aligns with the CAPABLE trial, where participants experienced significant reductions in insomnia severity and trends toward better overall sleep quality following a structured, 12‐week HIIT program. These findings are largely consistent with prior research indicating that physical activity can reduce the burden of sleep disturbances, improve overall quality of life, and potentially mitigate the long‐term effects of cancer and its treatment [43, 44]. Though there have been inconsistent results reported even among large meta‐analyses of exercise interventions and sleep health in oncology [45, 46, 47, 48]. The inconsistencies are likely attributed to differences in the types of exercise interventions included within the meta‐analysis (e.g., aerobic versus resistance‐based, low‐ and moderate‐ versus high‐intensity modalities) and/or the characteristics of patient populations (e.g., baseline sleep quality or symptoms, age, gender, race/ethnicity, cancer site and/or stage at diagnosis).

HIIT has emerged as a promising intervention for improving overall physical and psychological health, including enhanced cardiovascular fitness, metabolic function, and mental well‐being, alongside improvements in sleep quality, particularly in populations with chronic sleep disturbances like cancer survivors. Our results demonstrate that HIIT can significantly improve insomnia severity, as evidenced by reductions in ISI scores following a 12‐week HIIT program conducted three times per week. Furthermore, the PSQI showed a positive trend in overall sleep quality, although the change was not statistically significant. This suggests that HIIT may have a more targeted effect on specific sleep issues, such as insomnia, rather than global sleep quality. These findings are consistent with the notion that exercise, especially high‐intensity forms like HIIT, can help address the underlying physiological and psychological factors contributing to sleep disturbances.

There are several plausible mechanisms to explain the benefit of HIIT to sleep health that aren't mutually exclusive. It is possible that HIIT may improve sleep by positively influencing the sleep–wake cycle and circadian rhythms. Research indicates that regular high‐intensity exercise helps resynchronize circadian rhythms, a critical component of sleep regulation, which is often disrupted in cancer survivors due to treatment‐related factors [43]. In addition, HIIT has been shown to enhance metabolic health, reduce inflammation, and improve body composition—all of which contribute to better sleep quality. Lean body mass, in particular, correlates positively with non‐REM and slow‐wave sleep, which are considered restorative stages of sleep [44]. On the other hand, sarcopenia and obesity, characterized by low muscle and high fat mass, are associated with poorer sleep duration and efficiency [44, 49]. By increasing lean muscle mass and reducing fat, HIIT can potentially reverse these effects, improving sleep quality. Furthermore, HIIT can increase levels of BDNF, a neurotrophin essential for maintaining synaptic health, memory, and mood regulation. As previously mentioned, BDNF deficiencies are linked to poor sleep and metabolic imbalances. Studies suggest that exercise, particularly HIIT, significantly elevates BDNF levels, which could contribute to both improved mood and enhanced sleep quality [50]. Additionally, HIIT's effects on metabolism and weight loss may improve sleep by mitigating sleep‐disordered breathing (SDB) and improving overall sleep efficiency [51]. We hypothesize a similar physiology in both cancer and non‐cancer populations; however, there may be unique mechanisms at play in cancer that warrant further investigation. Beyond its physiological effects, HIIT offers several psychological benefits that are likely to contribute to improvements in sleep quality. Exercise, particularly in a structured group setting, provides emotional and social benefits that can reduce feelings of isolation, anxiety, and depression—factors known to worsen sleep disturbances [52]. The psychological impact of exercise is also thought to enhance sleep by promoting relaxation and reducing the physiological symptoms of stress, such as elevated cortisol levels [53]. By improving mood and reducing psychological distress, HIIT may provide cancer survivors with the emotional stability needed to improve sleep onset, quality, and duration.

### 4.1 Limitations and Future Directions

While the current studies provide strong evidence for the benefits of moderate‐ and high‐intensity physical activity, there are some limitations that warrant consideration. The primary limitation of Detroit ROCS cohort design is that it is observational so that determining whether the relationship between physical activity and sleep is a causal one is difficult due to the timing and nature of data collection. However, the similar benefit of HIIT on sleep health with the introduction of CAPABLE does support a causal association. Furthermore, Detroit ROCS relies on self‐reported physical activity using a well‐validated instrument. The CAPABLE trial offers improvement over self‐report in that participants were required to participate in HIIT training 3 h per week with the ability to assess adherence to the intervention. However, an objective measurement of participant exertion (i.e., wearable monitor) was not required. And while these short‐term results are encouraging, the long‐term effects of HIIT on sleep quality remain unclear. Future research should focus on evaluating the sustainability of these benefits over longer periods, understanding the barriers to long‐term changes in physical activity and understanding the underlying biology of the relationship. Future interventions may explore the potential for integration between HIIT and other interventions like cognitive behavioral therapy for sleep disturbances in cancer survivors.

## 5 Conclusion

In conclusion, the integration of HIIT into cancer survivorship care presents a promising, accessible intervention for improving sleep quality in cancer survivors. The multifactorial mechanisms behind HIIT's effectiveness contribute to its potential as an effective treatment for sleep disturbances. As evidence for HIIT's benefits continues to grow, it holds great promise for improving overall survivorship outcomes, particularly for underserved populations facing significant health disparities. Future research should focus on the long‐term sustainability of these benefits, as well as the exploration of tailored exercise programs to meet the diverse needs of cancer survivors.

## Author Contributions

**Grace E. Markey:** formal analysis (equal), writing – original draft (equal), writing – review and editing (equal). **Julie J. Ruterbusch:** conceptualization (equal), formal analysis (equal), supervision (equal), writing – original draft (equal), writing – review and editing (equal). **Tara E. Baird:** conceptualization (equal), supervision (equal). **Jennifer L. Martin:** conceptualization (equal), writing – review and editing (equal). **Ann G. Schwartz:** funding acquisition (equal), resources (equal), writing – original draft (equal), writing – review and editing (equal). **David G. Finlay:** methodology (equal), writing – review and editing (equal). **Trey Timban:** writing – review and editing (equal). **Matthew R. Trendowski:** writing – original draft (equal), writing – review and editing (equal). **M. Safwan Badr:** conceptualization (equal), writing – review and editing (equal). **Kerri Winters‐Stone:** conceptualization (equal), writing – review and editing (equal). **Jennifer L. Beebe‐Dimmer:** conceptualization (equal), formal analysis (equal), funding acquisition (equal), investigation (equal), methodology (equal), project administration (equal), resources (equal), supervision (equal), writing – original draft (equal), writing – review and editing (equal).

## Funding

This work was supported by the National Cancer Institute at the National Institutes of Health (U01 CA199240; Supplement), the Epidemiology Research Core, and the National Cancer Institute Center Grant (P30 CA022453) awarded to the Barbara Ann Karmanos Cancer Institute at Wayne State University. CAPABLE funding sources: National Institutes of Health (NIH), National Cancer Institute (NCI), Michigan Department of Health and Human Services (MDHHS) and the Barbara Ann Karmanos Cancer Institute (Population Studies & Disparities Research Program internal funds and Strategic Research Initiative Grants (SRIG)).

## Conflicts of Interest

The authors declare no conflicts of interest.

## Data Availability

The data that support the findings of this study are available to investigators by request at https://detroitrocs.org/dnn/For‐Researchers.
